# May chronic cough in chronic obstructive pulmonary disease be a contraindication of Percutaneous Endoscopic Gastrostomy placement: a case report

**DOI:** 10.1186/s12876-021-01603-0

**Published:** 2021-01-21

**Authors:** A. G. Gravina, A. Tessitore, V. M. Ormando, F. Nagar, M. Romeo, M. R. Amato, M. Dallio, C. Loguercio, A. Federico, M. Romano, F. Ferraro

**Affiliations:** 1 Hepatogastropenterology Unit, Department of Precision Medicine, University of Campania “Luigi Vanvitelli”, Via Pansini, 5, 80131 Naples, Italy; 2 Department of Medical, Surgical, Neurological, Metabolic and Geriatric Sciences, University of Campania “Luigi Vanvitelli”, Naples, Italy; 3 Department of Woman, Child and General and Specialized Surgery, University of Campania “Luigi Vanvitelli”, Naples, Italy

**Keywords:** Percutaneous endoscopic gastrostomy, Buried bumper syndrome, Chronic obstructive pulmonary disease, Cough

## Abstract

**Background:**

Percutaneous Endoscopic Gastrostomy (PEG) can involve some complications, despite the good safety of its track record. The Buried Bumper Syndrome (BBS) is a rare, late and dangerous complication that consists in the erosion of the internal bumper through the gastric wall.

Case presentation

We report the development of BBS in a man with chronic obstructive pulmonary disease (COPD) who had a persistent chronic cough which was prevalently but not solely in the morning and required placement of a PEG tube for continuous infusion of Levodopa/carbidopa intestinal gel for advanced Parkinson's disease.

**Conclusion:**

We believe that COPD with chronic cough while not representing an absolute contraindication to PEG placement, may potentially cause BBS and therefore an appropriate regimen of tube care by expert personnel is mandatory in this setting.

## Background

Percutaneous endoscopic gastrostomy (PEG) is considered a safe procedure to provide long term enteral nutrition and enteral access [1]. In addition, the PEG tube can be used to intubate the proximal jejunum with an extension tube (J-PEG) to allow the pharmacological treatment of Advanced Parkinson’s Disease through a continuous delivery of levodopa (L-Dopa)/carbidopa intestinal gel (LCIG). It represents a new approach in patients with Advanced Parkinson’s Disease who respond to oral administration of L-Dopa therapy but with dyskinesias and motor fluctuations which are poorly managed by optimized oral therapy alone [2].

Although it has been generally considered safe, PEG tube placement can be associated with many potential complications that can be classified as minor or major. Major complications include aspiration pneumonia, hemorrhage, buried bumper syndrome (BBS), colon perforation during the PEG insertion, necrotizing fasciitis [1, 3–5]. An unusual and late complication could be the BBS that occurs when the internal bumper erodes from the lumen of the stomach into the gastric wall or subcutaneous tissue. BBS is due to excessive tension between the internal and external bumpers that causes ischemic necrosis of the gastric wall. It usually manifests with the signs and symptoms of infection due to leakage of gastric content from around the PEG tube insertion site in the peristomal site. We may observe fixity of the PEG tube, abdominal pain and resistance to water or nutritional solution infusion. Inability to insert PEG tube, loss of patency and leakage around the PEG tube are considered to be a typical symptomatic triad [6]. However, if the tube is not removed as soon as possible, severe complications might happen such as perforation of the stomach, peritonitis and death [7–9].

## Case presentation

**Fig. 1:**
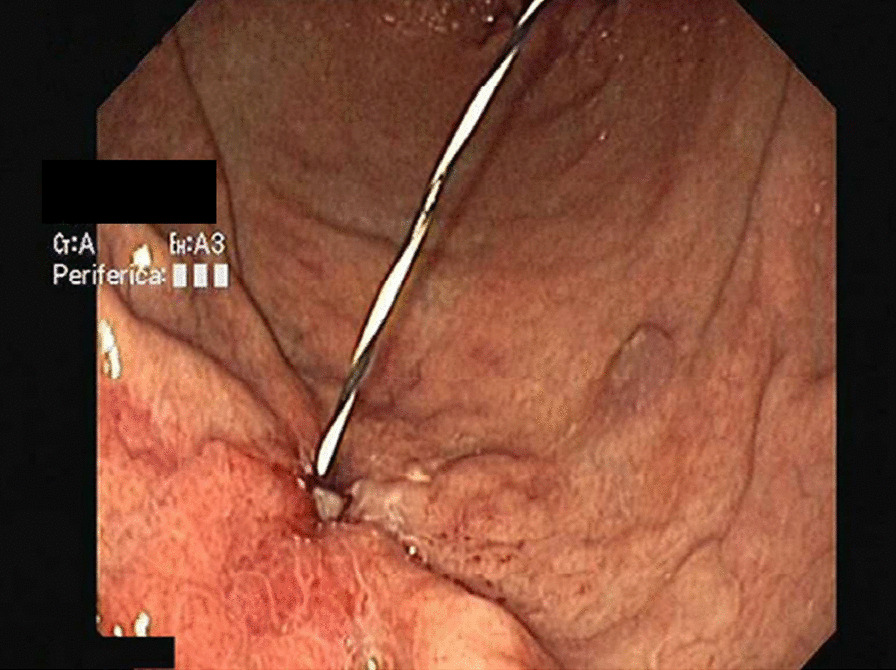
Endoscopic image of BBS, the internal bumper is not visible on the gastric wall

**Fig. 2:**
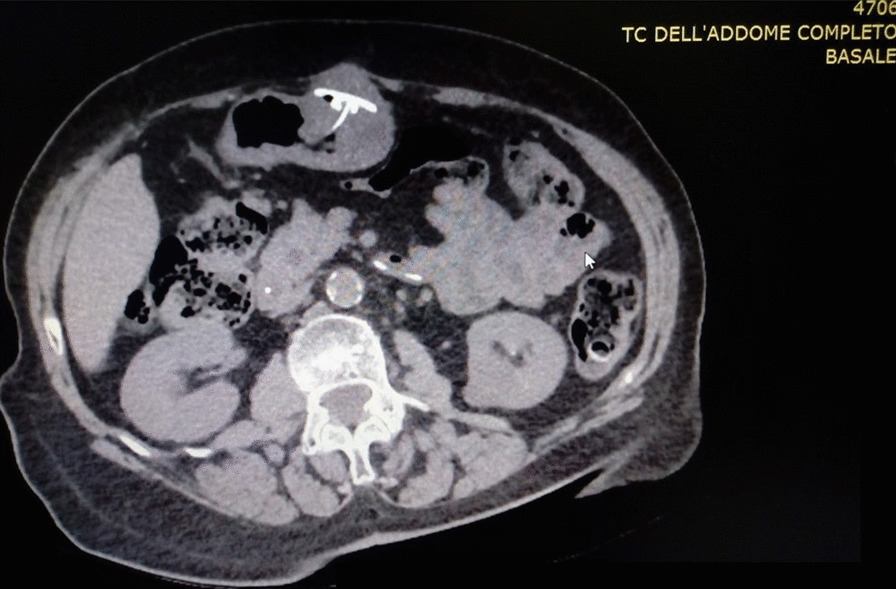
Computed Tomography (CT) image demonstrating a BBS type 3

A 57 years old man affected by Advanced Parkinson’s Disease was selected for J-PEG placement to allow the continuous administration of LCIG in duodenum (AbbVie PEG polyurethane Tube 20 F). We obtained informed consent. He had a smoking history of 30/40 cigarettes per day for the past 30 years reporting a chronic cough, prevalently but not solely in the morning. On examination he appeared overweight (BMI = 29) with an abdominal fat distribution. Lung clinical examination revealed a barrel chest decreased brief sounds, with moderate inspiratory and expiratory wheezing, he used accessory muscles of respiration. There were signs and symptoms of chronic obstructive pulmonary disease (COPD); spirometry showed a ratio of forced expiratory volume in 1s (FEV1) and forced vital capacity (FVC) slightly lower than normal (65%); and low maximum expiratory flow (MEF was 80% at 25% and 50%). There were no absolute contraindication to place PEG tube. A pull-technique was used and a routine post insertion endoscopic confirmation of satisfactory PEG placement (AbbVie PEG polyurethane Tube 20 F) was performed at the end of the procedure. The PEG appeared successfully placed. Thirteen days later the tube was unable to infuse solution, there was a peritubal leak and the patient had abdominal pain with signs of edema and erythema of the tube insertion area. On the clinical examination we observed that during cough attack abdomen of our patient become prominent. Endoscopic evaluation showed that gastric mucosa covering internal gastrostomy site resulted in a complete closure of the orifice with visualization of only the J-tube extension. The internal bumper was not visible on the gastric wall but mucosa was ulcerated at the presumed site (Fig. 1). The patient underwent computed tomography (CT) (Fig. 2) of the abdomen, BBS was recognized and the device was removed by using a needle-knife assisted endoscopic dissection technique. After one week, following a thorough multidisciplinary evaluation due to the patient’s considerable anesthesiological risk, we positioned a new PEG-tube endoscopically in an area at a distance from the one used previously. Unfortunately, however, one month later the BBS presented again..Because the internal bumper was not visible endoscopically due to its dislocation into the abdominal wall, a microinvasive endoscopic needle knife-assisted approach was not feasible, and, therefore, we removed it surgically.

## Discussion and conclusion

BBS is usually a late and rare complication of PEG occurring in 0.3–2.4% of the patients [1]. BBS usually appears no earlier than 4 months after PEG placement. Nevertheless it has been described a case of BBS after three weeks from tube placement [9]. In our patient, BBS occurred thirteen days after PEG insertion and then again after one month. The case of our patient suggests that COPD and chronic cough may represent a potential cause of BBS, even though the association between COPD with chronic cough and BBS as in our case does not necessarily imply a cause-effect relationship. Our case report underlines the important role of the traction performed by the respiratory movements in a patient with COPD with little airway obstruction, as evaluated by spirometry. The persistent cough produced a traction of the PEG tube and a bumper compression which in turn lead to gastric wall erosion. We cannot completely rule out the possibility that too much tension was placed on the bumper during PEG tube insertion or that there had been a lack of appropriate management of the tube. The first time, after thirteen days from the first positioning, there was a displacement corresponding to a type 3 BBS because the internal bumper appeared total visible at mobilization. The second time, after one month from the second PEG placement, a deep type 4 BBS occurred. This is according to Richter-Schraq HJ et al. who described 4 types of BBS: in type 1 the internal bumper is outside the body or in the subcutaneous tissue; in type 2 the internal bumper is partially visible in gastric lumen; in type 3 the internal bumper is not visible in the gastric lumen and is in the most superficial layers of the gastric wall; in type 4 the internal bumper is not visible in the gastric cavity and is in the deeper layers of the gastric wall [7]. BBS is a dangerous major complication of the PEG placement because it may cause infection, necrotizing fasciitis [3, 10], peritonitis and consequently septic shock with a fatal outcome [8]. Absolute contraindication to PEG placement are pharyngeal and esophageal occlusion for pull technique, active serious coagulopathy, hemodynamic instability, sepsis, severe ascites, peritonitis, peritoneal carcinomatosis, portal hypertension with gastric varices, total gastrectomy [1, 11]. Our patient had none of these absolute contraindications. Moreover some studies associated COPD at a higher risk of fatal outcome due to an increased susceptibility of COPD patients to develop gastroesophageal reflux disease that is strictly associated to a higher aspiration pneumonia risk, rather than a higher risk of BBS rising [12]. We believe that COPD with chronic cough may cause the displacement of the internal bumper thus causing BBS. Therefore, in case of a complication such as BBS it is important to have a radiologic evaluation in order to better assess the problem and possibly solving it through a surgical approach.

PEG is a safe for enteral nutrition and J-PEG has a fundamental role in the pharmacological treatment of Advanced Parkinson’s Disease as well as to ability to feed directly into the jejunum. PEG tube placement may have complications and BBS is one of these ones. PEG-related complications are mostly prevented by an appropriate tube care.. It is essential for all patients, but particularly in those who have a chronic cough, that during the daily tube cleaning the PEG is not only rotated, but advanced further into the stomach and then pulled back until set to the correct length and tension. In these patients, the tube needs to have some slack to accommodate the excursion of the abdominal wall during forced expiration while coughing. In order to avoid BBS, some PEG tubes with externally removable internal bumpers were found useful in the treatment of BBS allowing the bumper removal by external traction without any endoscopic or surgical methods [13].

COPD with chronic cough does not represent a contraindication to placement of PEG tube but in our opinion, by facilitating movements of internal bumper, it may favor erosion of internal bumper from gastric lumen into the gastric wall or subcutaneous tissue thus causing BBS. Therefore, based on this case report, not only much attention should be paid to PEG placement but also an accurate post-procedure tube care should be strongly advised in this particular setting.

## Data Availability

All the data supporting our findings can be found in the patient hospital medical report. Unlikely, we don't have an electronic repository at out Institution. All the personal patients' data, including biochemical, endoscopic and radiological exams, are stored on paper at our hospital. Therefore we can't share our data.

## Abbreviations

PEG: Percutaneous endoscopic gastrostomy
BBS: Buried bumper syndrome
COPD: Chronic obstructive pulmonary disease
J-PEG: PEG with jejunal extension tube
L-Dopa: Levodopa
LCIG: Levodopa/carbidopa intestinal gel
BMI: Body max index
FEV1: Forced expiratory volume in 1s
FVC: Forced vital capacity
MEF: Maximum expiratory flow
CT: Computed tomography
